# *N*′-[Bis(benzyl­sulfan­yl)methyl­idene]-4-meth­oxy­benzohydrazide

**DOI:** 10.1107/S1600536810025389

**Published:** 2010-07-03

**Authors:** Jerry P. Jasinski, Ray J. Butcher, S. K. Kushawaha, M. K. Bharty, N. K. Singh

**Affiliations:** a Department of Chemistry, Keene State College, 229 Main Street, Keene, NH 03435-2001, USA; b Department of Chemistry, Howard University, 525 College Street NW, Washington, DC 20059, USA; c Department of Chemistry, Banaras Hindu University, Varanasi 221 005, India

## Abstract

In the title compound, C_23_H_22_N_2_O_2_S_2_, the dihedral angles between the 4-meth­oxy-substituted phenyl ring and the other two phenyl rings are 84.4 (4) and 77.7 (1)°, respectively, while the dihedral angle between the two phenyl rings is 57.5 (2)°. The amino group is not involved in an N—H hydrogen bond. The crystal packing is established by inter­molecular C—H⋯O packing inter­actions involving a relatively rare weak three-center hydrogen bond between the keto O atom and H atoms of the two nearby phenyl rings, which link the mol­ecules into chains running along the *a* axis. Additional weak inter­molecular hydrogen-bond inter­actions between the 4-meth­oxy O atom and one of the phenyl rings and provide added stability to the crystal packing.

## Related literature

For radiopharmaceutical applications of dithio­carbazate derivatives, see: Boschi *et al.* (2003 ▶). For dithio­carbazate derivatives as anti­cancer and anti­microbial drugs, see: Bharti *et al.* (2000 ▶). For a related structure, see: Singh *et al.* (2007 ▶).
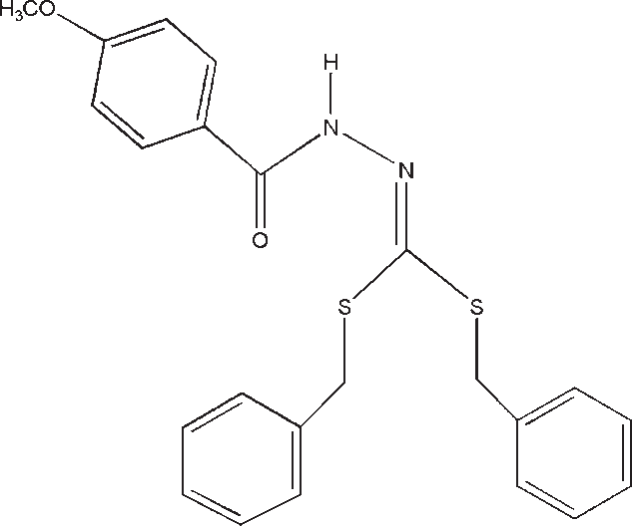


## Experimental

### 

#### Crystal data

C_23_H_22_N_2_O_2_S_2_
*M* *_r_* = 422.55
Triclinic, 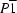

*a* = 9.838 (2) Å
*b* = 9.845 (2) Å
*c* = 11.307 (2) Å
α = 70.25 (3)°
β = 90.00 (3)°
γ = 89.37 (3)°
*V* = 1030.6 (4) Å^3^
*Z* = 2
Mo *K*α radiation
μ = 0.28 mm^−1^
*T* = 100 K
0.30 × 0.26 × 0.22 mm

#### Data collection

Bruker APEX CCD area-detector diffractometer
Absorption correction: multi-scan (*SADABS*; Sheldrick, 1996 ▶) *T*_min_ = 0.921, *T*_max_ = 0.941
12424 measured reflections
6210 independent reflections
5954 reflections with *I* > 2σ(*I*)
*R*_int_ = 0.015

#### Refinement

*R*[*F* ^2^ > 2σ(*F* ^2^)] = 0.037
*wR*(*F* ^2^) = 0.098
*S* = 1.06
6210 reflections
263 parameters
H-atom parameters constrained
Δρ_max_ = 0.56 e Å^−3^
Δρ_min_ = −0.35 e Å^−3^

### 

Data collection: *APEX2* (Bruker, 2006 ▶); cell refinement: *SAINT* (Bruker, 2006 ▶); data reduction: *SAINT*; program(s) used to solve structure: *SHELXS97* (Sheldrick, 2008 ▶); program(s) used to refine structure: *SHELXL97* (Sheldrick, 2008 ▶); molecular graphics: *SHELXTL* (Sheldrick, 2008 ▶); software used to prepare material for publication: *SHELXTL* and *PLATON* (Spek, 2009 ▶).

## Supplementary Material

Crystal structure: contains datablocks global, I. DOI: 10.1107/S1600536810025389/bt5283sup1.cif

Structure factors: contains datablocks I. DOI: 10.1107/S1600536810025389/bt5283Isup2.hkl

Additional supplementary materials:  crystallographic information; 3D view; checkCIF report

## Figures and Tables

**Table 1:** Hydrogen-bond geometry (Å, °)

*D*—H⋯*A*	*D*—H	H⋯*A*	*D*⋯*A*	*D*—H⋯*A*
C6*A*—H6*AA*⋯O1^i^	0.95	2.41	3.2516 (17)	147
C1*B*—H1*BA*⋯O1^ii^	0.99	2.35	3.2872 (16)	157
C3*B*—H3*BA*⋯O1^iii^	0.95	2.67	3.5351 (18)	152
C6*B*—H6*BA*⋯O2^iv^	0.95	2.47	3.4173 (17)	174

Symmetry codes: (i) 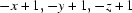; (ii) 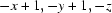; (iii) 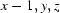; (iv) 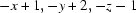.

**Fig. 1.**
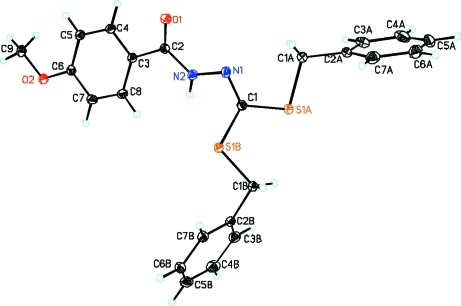
Molecular structure of C23H22N2O2S2, showing the atom labeling scheme and 50% probability displacement ellipsoids.

**Fig. 2.**
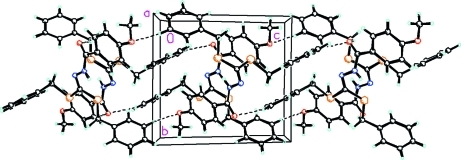
Packing diagram of C23H22N2O2S2,, viewed down the b axis. Dashed lines indicate weak intermolecular C—H···O hydrogen bonding interactions.

## supplementary crystallographic information

### Comment

Dithiocarbazate derivatives have been widely studied in radiopharmaceutical applications (Boschi *et al.*, 2003) and have potential biological activity as anticancer and antimicrobial drugs (Bharti *et al.*, 2000). This functional group is of particular interest and can coordinate to metals to give structures with different geometries and properties. As a part of our ongoing research on the dithio derivatives of acid hydrazides, we report here the crystal structure of the title compound, C_23_H_22_N_2_O_2_S_2_, a new 4-methoxy benzoic acid bis benzyl sulfanyl methylene hydrazide with a relatively rare three-center donor hydrogen bond.

The sum of the angles around C1 (120.00 (8)°) and the S1A/C1/S1B bond angle of 117.52 (7)° indicated nearly planar *sp*^2^ hybridized behavior (Fig. 1). The molecule can be divided into three distinct fragments with regard to their spatial orientation *viz* keto-amide, 4-methoxy benzene and bis benzyl sulfanyl methylene groups. Regarding the keto-amide group, all three rings including the 4-methoxy benzene, and two separate benzyl rings [A & B] have their mean planes twisted by 25,7(7)°, [B] 69.1 (6)° and [A] 76.5 (8)°, respectively. The dihedral angle between the 4-methoxy benzene group and two nearby benzyl groups is [B] 84.4 (4)° and [A] 77.7 (0)°, respectively. The mean plane between the two separate benzyl groups [A & B] is 57.5 (2)°. The torsion angles around the keto-amide linkage provides geometric support to these twist angles (C1/N1/N2/C2 = 154.32 (10)°; O1/C2/C3/C4 = 151.22 (11)°; N2/N1/C1/S1A = -173.64 (7)°; N/2/N/1/C1/S1B = 6.23 (13)°). The mean planes of the two sulfanyl groups are twisted nearly perpendicular to the dihedral planes of their adjacent benzyl rings with angles of [A] 70.6 (7)° and [B] 77.6 (4)° separating their groups to avoid steric hindrance. The N1 atom in the amide linkage possesses distorted tetrahedral geometry (C1/N1/N2 = 112.66 (10)°) while the N2 atom lies in a more planar fashion (N1/N2/C2/O1 = -1.25 (17)°). The C1—N1 and C2—N2 bond lengths (1.29253 (14)Å and 1.3574 (14) Å) lie between typical C—N and C═N values owing to the extensive delocalization of π electron density over the C2/N2/N1/C1 linkage.

A relatively rare weak three-center hydrogen bond configuration and additional weak C–H···O donor hydrogen bonds can be seen linking the molecules into chains along the (011) plane (Fig. 2). Additional hydrogen bonds between the 4-methoxy oxygen atom (O2) and one of the benzyl groups (C6B–H6BA···O2) help to stabilize crystal packing.

### Experimental

The potassium salt of *N*'-(4-methoxy benzoyl) hydrazine carbodithioate was prepared by adding carbon disulfide (0.04 mol, 2.4 ml) to a solution of 4-methoxy benzoic acid hydrazide(0.02 mol, 3.32 g) and potassium hydroxide (0.02 mol, 1.12 g) in methanol (30 ml) then stirring the reaction mixture for 2 h. The solid separated was filtered off, washed with 10% (*v*/*v*) mixture of ethanol-ether and dried *in vacuo*. Yield 1.54 g, 55%, m.p. 518 K. The title compound was prepared by drop wise addition of benzyl chloride (0.02 mol, 2.53 g) to a suspension of potassium salt of *N*'-(4-methoxy benzoyl)hydrazine carbodithioate (0.01 mol, 2.80 g) in methanol (20 ml) and stirring the reaction mixture for a period of 5–6 h. The reaction mixture was filtered and the solution was evaporated almost to dryness. The solid was washed several times with carbon tetrachloride and then with chloroform and recrystallized from methanol. Transparent white shining crystals of (I) (m.p. 475 K), suitable for X-ray analysis were obtained by slow evaporation of the methanol solution over a period of 9–10 days (yield 2.53 g, 60%): Anal Calcd (%): C, 65.31; H, 5.20; N, 6.62; S, 15.17; Found(%) for C_23_H_22_N_2_O_2_S_2_ (422.55): C, 65.52; H, 5.15; N, 6.75; S, 15.30.

### Refinement

All of the H atoms were placed in their calculated positions and then refined using the riding model with C–H = 0.95–0.99 Å, N–H = 0.88\|A%, with *U*_iso_(H) = 1.18–1.49*U*_eq_(C) and *U*_iso_(H) = 1.19*U*_eq_(N).

### Crystal data

**Table:**

C_23_H_22_N_2_O_2_S_2_	*Z* = 2
*M**_r_* = 422.55	*F*(000) = 444
Triclinic, *P*1	*D*_x_ = 1.362 Mg m^−^^3^
Hall symbol: -P 1	Mo *K*α radiation, λ = 0.71073 Å
*a* = 9.838 (2) Å	Cell parameters from 5412 reflections
*b* = 9.845 (2) Å	θ = 2.4–30.5°
*c* = 11.307 (2) Å	µ = 0.28 mm^−^^1^
α = 70.25 (3)°	*T* = 100 K
β = 90.00 (3)°	Chunk, colorless
γ = 89.37 (3)°	0.30 × 0.26 × 0.22 mm
*V* = 1030.6 (4) Å^3^	

### Data collection

**Table:**

Bruker APEX CCD area-detector diffractometer	6210 independent reflections
Radiation source: fine-focus sealed tube	5954 reflections with *I* > 2σ(*I*)
graphite	*R*_int_ = 0.015
Detector resolution: 8.33 pixels mm^-1^	θ_max_ = 30.5°, θ_min_ = 1.9°
φ and ω scans	*h* = −14→14
Absorption correction: multi-scan (*SADABS*; Sheldrick, 1996)	*k* = −14→14
*T*_min_ = 0.921, *T*_max_ = 0.941	*l* = −16→16
12424 measured reflections	

### Refinement

**Table:**

Refinement on *F*^2^	Primary atom site location: structure-invariant direct methods
Least-squares matrix: full	Secondary atom site location: difference Fourier map
*R*[*F*^2^ > 2σ(*F*^2^)] = 0.037	Hydrogen site location: inferred from neighbouring sites
*wR*(*F*^2^) = 0.098	H-atom parameters constrained
*S* = 1.06	*w* = 1/[σ^2^(*F*_o_^2^) + (0.0512*P*)^2^ + 0.4754*P*] where *P* = (*F*_o_^2^ + 2*F*_c_^2^)/3
6210 reflections	(Δ/σ)_max_ = 0.001
263 parameters	Δρ_max_ = 0.56 e Å^−^^3^
0 restraints	Δρ_min_ = −0.35 e Å^−^^3^

### Special details

**Table:**

Experimental. Spectroscopic analysis: IR(KBr, ν cm^-1^): 3285, (–NH); 1664, (C=O); 1604, (Thioamide I[β(NH + ν(CN)]; 1309, (Thioamide II [ν(CN) + β(NH)]; 760, (Thioamide IV, ν(C—S); 1068 (N—N). ^1^H NMR (CDCl_3_, δ, p.p.m.): 1.80, (s, 3H, OCH_3_); 4.25(d, 4H, –CH_2_); 9.40, (s, 1H, NH); 6.90, (m,5*H*, phenyl ring); 7.35 – 7.46, (m,10*H*, –CH_2_Ph); ^13^C NMR (CDCl_3_, δ, p.p.m.): 193.14, (C—S); 162.40, (C=O); 136.66, (C4,8); 115.92, (C5,7); 125.03, (C6); 128.37, (C3); 127.84, (C2A); 112.34, (C3A,7 A); 129.76, (C4A,6 A); 127.78, (C5A); 55.31,(CH_2_); 36.66, (CH_3_).
Geometry. All e.s.d.'s (except the e.s.d. in the dihedral angle between two l.s. planes) are estimated using the full covariance matrix. The cell e.s.d.'s are taken into account individually in the estimation of e.s.d.'s in distances, angles and torsion angles; correlations between e.s.d.'s in cell parameters are only used when they are defined by crystal symmetry. An approximate (isotropic) treatment of cell e.s.d.'s is used for estimating e.s.d.'s involving l.s. planes.
Refinement. Refinement on *F*^2^ against ALL reflections. Weighted *R*-factors *wR* and all goodnesses of fit *S* are based on *F*^2^, conventional *R*-factors *R* are based on *F*, with *F* set to zero for negative *F*^2^. The threshold expression of *F*^2^ > σ(*F*^2^) is used only for calculating *R*-factors(gt) *etc*. and is not relevant to the choice of reflections for refinement. *R*-factors based on *F*^2^ are statistically about twice as large as those based on *F*, and *R*- factors based on ALL data will be even larger.

### Fractional atomic coordinates and isotropic or equivalent isotropic displacement parameters (Å^2^)

**Table:**

	*x*	*y*	*z*	*U*_iso_*/*U*_eq_	
S1A	0.34589 (3)	0.41786 (3)	0.19374 (2)	0.01665 (7)	
S1B	0.33079 (3)	0.67558 (3)	−0.04284 (2)	0.01674 (7)	
O1	0.79463 (8)	0.72953 (9)	0.08230 (8)	0.01885 (16)	
O2	0.78658 (9)	1.34644 (9)	−0.34225 (8)	0.01967 (16)	
N1	0.53203 (9)	0.62306 (10)	0.12649 (9)	0.01678 (17)	
N2	0.56651 (9)	0.75955 (10)	0.04495 (9)	0.01679 (17)	
H2A	0.5015	0.8188	0.0037	0.020*	
C1	0.41847 (11)	0.57822 (11)	0.09652 (10)	0.01510 (18)	
C2	0.69749 (11)	0.80369 (11)	0.02737 (10)	0.01495 (18)	
C3	0.71432 (10)	0.95034 (11)	−0.06794 (10)	0.01506 (18)	
C4	0.62403 (11)	1.01134 (12)	−0.16830 (11)	0.0182 (2)	
H4A	0.5440	0.9612	−0.1749	0.022*	
C5	0.65070 (11)	1.14405 (12)	−0.25784 (11)	0.0190 (2)	
H5A	0.5886	1.1852	−0.3252	0.023*	
C6	0.76938 (11)	1.21779 (11)	−0.24914 (10)	0.01585 (19)	
C7	0.86039 (11)	1.15852 (12)	−0.14981 (10)	0.01734 (19)	
H7A	0.9410	1.2080	−0.1436	0.021*	
C8	0.83092 (11)	1.02579 (12)	−0.06019 (10)	0.01714 (19)	
H8A	0.8920	0.9855	0.0081	0.021*	
C9	0.90880 (12)	1.42350 (13)	−0.33998 (11)	0.0214 (2)	
H9A	0.9094	1.5135	−0.4120	0.032*	
H9B	0.9878	1.3639	−0.3445	0.032*	
H9C	0.9128	1.4458	−0.2619	0.032*	
C1A	0.46244 (12)	0.37100 (16)	0.32723 (11)	0.0269 (3)	
H1AA	0.5418	0.3175	0.3106	0.032*	
H1AB	0.4957	0.4602	0.3388	0.032*	
C2A	0.39201 (11)	0.27942 (12)	0.44481 (10)	0.0182 (2)	
C3A	0.37413 (13)	0.13247 (13)	0.47003 (13)	0.0242 (2)	
H3AA	0.4041	0.0887	0.4114	0.029*	
C4A	0.31194 (15)	0.04925 (15)	0.58191 (14)	0.0337 (3)	
H4AA	0.2996	−0.0512	0.5992	0.040*	
C5A	0.26837 (14)	0.11268 (19)	0.66751 (13)	0.0371 (4)	
H5AA	0.2267	0.0557	0.7437	0.045*	
C6A	0.28544 (14)	0.25882 (19)	0.64225 (12)	0.0331 (3)	
H6AA	0.2554	0.3025	0.7009	0.040*	
C7A	0.34638 (13)	0.34147 (14)	0.53129 (12)	0.0244 (2)	
H7AA	0.3571	0.4421	0.5141	0.029*	
C1B	0.17073 (11)	0.58242 (12)	−0.03828 (10)	0.01767 (19)	
H1BA	0.1868	0.4789	−0.0253	0.021*	
H1BB	0.1117	0.5909	0.0298	0.021*	
C2B	0.10777 (11)	0.65860 (11)	−0.16520 (10)	0.01608 (19)	
C3B	0.00363 (12)	0.76019 (13)	−0.17959 (12)	0.0214 (2)	
H3BA	−0.0315	0.7787	−0.1082	0.026*	
C4B	−0.04888 (13)	0.83455 (14)	−0.29857 (13)	0.0277 (3)	
H4BA	−0.1206	0.9029	−0.3081	0.033*	
C5B	0.00326 (14)	0.80896 (14)	−0.40303 (12)	0.0287 (3)	
H5BA	−0.0324	0.8602	−0.4842	0.034*	
C6B	0.10777 (13)	0.70825 (14)	−0.38928 (11)	0.0249 (2)	
H6BA	0.1440	0.6915	−0.4611	0.030*	
C7B	0.15918 (12)	0.63218 (12)	−0.27049 (11)	0.0194 (2)	
H7BA	0.2293	0.5622	−0.2610	0.023*	

### Atomic displacement parameters (Å^2^)

**Table:**

	*U*^11^	*U*^22^	*U*^33^	*U*^12^	*U*^13^	*U*^23^
S1A	0.01578 (12)	0.01722 (12)	0.01415 (12)	−0.00345 (9)	−0.00047 (9)	−0.00155 (9)
S1B	0.01535 (12)	0.01657 (12)	0.01539 (12)	−0.00216 (9)	−0.00130 (9)	−0.00152 (9)
O1	0.0167 (4)	0.0186 (4)	0.0182 (4)	0.0004 (3)	−0.0014 (3)	−0.0023 (3)
O2	0.0205 (4)	0.0164 (4)	0.0183 (4)	−0.0038 (3)	−0.0003 (3)	−0.0007 (3)
N1	0.0166 (4)	0.0150 (4)	0.0168 (4)	−0.0024 (3)	0.0016 (3)	−0.0026 (3)
N2	0.0145 (4)	0.0142 (4)	0.0188 (4)	−0.0014 (3)	−0.0007 (3)	−0.0018 (3)
C1	0.0156 (4)	0.0151 (4)	0.0136 (4)	0.0003 (3)	0.0003 (3)	−0.0036 (3)
C2	0.0156 (4)	0.0156 (4)	0.0139 (4)	−0.0018 (3)	0.0008 (3)	−0.0052 (4)
C3	0.0143 (4)	0.0146 (4)	0.0155 (4)	−0.0004 (3)	0.0000 (3)	−0.0040 (4)
C4	0.0164 (5)	0.0185 (5)	0.0182 (5)	−0.0026 (4)	−0.0030 (4)	−0.0042 (4)
C5	0.0180 (5)	0.0193 (5)	0.0172 (5)	−0.0007 (4)	−0.0039 (4)	−0.0028 (4)
C6	0.0168 (4)	0.0145 (4)	0.0153 (4)	−0.0002 (3)	0.0014 (3)	−0.0037 (4)
C7	0.0152 (4)	0.0171 (5)	0.0184 (5)	−0.0022 (4)	−0.0005 (4)	−0.0041 (4)
C8	0.0145 (4)	0.0177 (5)	0.0174 (5)	−0.0004 (4)	−0.0021 (4)	−0.0036 (4)
C9	0.0204 (5)	0.0198 (5)	0.0215 (5)	−0.0054 (4)	0.0032 (4)	−0.0034 (4)
C1A	0.0185 (5)	0.0365 (7)	0.0165 (5)	−0.0083 (5)	−0.0030 (4)	0.0032 (5)
C2A	0.0152 (4)	0.0204 (5)	0.0146 (4)	−0.0019 (4)	−0.0026 (4)	−0.0003 (4)
C3A	0.0221 (5)	0.0212 (5)	0.0283 (6)	0.0004 (4)	−0.0042 (4)	−0.0071 (5)
C4A	0.0268 (6)	0.0224 (6)	0.0393 (8)	−0.0069 (5)	−0.0087 (5)	0.0061 (5)
C5A	0.0208 (6)	0.0525 (9)	0.0213 (6)	−0.0036 (6)	−0.0004 (5)	0.0096 (6)
C6A	0.0250 (6)	0.0549 (9)	0.0175 (5)	0.0098 (6)	−0.0031 (4)	−0.0101 (6)
C7A	0.0251 (6)	0.0260 (6)	0.0212 (5)	0.0043 (4)	−0.0073 (4)	−0.0071 (4)
C1B	0.0163 (4)	0.0185 (5)	0.0159 (4)	−0.0033 (4)	−0.0008 (4)	−0.0027 (4)
C2B	0.0145 (4)	0.0161 (4)	0.0158 (4)	−0.0029 (3)	−0.0009 (3)	−0.0030 (4)
C3B	0.0173 (5)	0.0220 (5)	0.0237 (5)	0.0004 (4)	−0.0003 (4)	−0.0064 (4)
C4B	0.0219 (5)	0.0234 (6)	0.0324 (6)	0.0024 (4)	−0.0083 (5)	−0.0026 (5)
C5B	0.0304 (6)	0.0262 (6)	0.0224 (6)	−0.0083 (5)	−0.0101 (5)	0.0014 (5)
C6B	0.0291 (6)	0.0287 (6)	0.0165 (5)	−0.0110 (5)	0.0006 (4)	−0.0068 (4)
C7B	0.0181 (5)	0.0212 (5)	0.0193 (5)	−0.0037 (4)	0.0013 (4)	−0.0075 (4)

### Geometric parameters (Å, °)

**Table:**

S1A—C1	1.7500 (13)	C1A—H1AB	0.9900
S1A—C1A	1.8231 (14)	C2A—C7A	1.3887 (17)
S1B—C1	1.7626 (13)	C2A—C3A	1.3899 (17)
S1B—C1B	1.8230 (12)	C3A—C4A	1.399 (2)
O1—C2	1.2289 (14)	C3A—H3AA	0.9500
O2—C6	1.3582 (14)	C4A—C5A	1.383 (2)
O2—C9	1.4336 (14)	C4A—H4AA	0.9500
N1—C1	1.2923 (14)	C5A—C6A	1.381 (2)
N1—N2	1.3941 (13)	C5A—H5AA	0.9500
N2—C2	1.3574 (14)	C6A—C7A	1.384 (2)
N2—H2A	0.8800	C6A—H6AA	0.9500
C2—C3	1.4923 (15)	C7A—H7AA	0.9500
C3—C8	1.3920 (15)	C1B—C2B	1.5060 (16)
C3—C4	1.4019 (16)	C1B—H1BA	0.9900
C4—C5	1.3836 (16)	C1B—H1BB	0.9900
C4—H4A	0.9500	C2B—C3B	1.3942 (16)
C5—C6	1.4025 (16)	C2B—C7B	1.3954 (16)
C5—H5A	0.9500	C3B—C4B	1.3925 (18)
C6—C7	1.3961 (16)	C3B—H3BA	0.9500
C7—C8	1.3899 (16)	C4B—C5B	1.386 (2)
C7—H7A	0.9500	C4B—H4BA	0.9500
C8—H8A	0.9500	C5B—C6B	1.392 (2)
C9—H9A	0.9800	C5B—H5BA	0.9500
C9—H9B	0.9800	C6B—C7B	1.3902 (17)
C9—H9C	0.9800	C6B—H6BA	0.9500
C1A—C2A	1.5047 (17)	C7B—H7BA	0.9500
C1A—H1AA	0.9900		
			
C1—S1A—C1A	100.40 (6)	C7A—C2A—C3A	119.11 (11)
C1—S1B—C1B	106.17 (6)	C7A—C2A—C1A	119.74 (11)
C6—O2—C9	117.42 (9)	C3A—C2A—C1A	121.13 (12)
C1—N1—N2	112.66 (10)	C2A—C3A—C4A	119.85 (13)
C2—N2—N1	121.87 (9)	C2A—C3A—H3AA	120.1
C2—N2—H2A	119.1	C4A—C3A—H3AA	120.1
N1—N2—H2A	119.1	C5A—C4A—C3A	120.22 (13)
N1—C1—S1A	120.92 (9)	C5A—C4A—H4AA	119.9
N1—C1—S1B	121.56 (9)	C3A—C4A—H4AA	119.9
S1A—C1—S1B	117.52 (7)	C6A—C5A—C4A	119.99 (13)
O1—C2—N2	123.68 (10)	C6A—C5A—H5AA	120.0
O1—C2—C3	122.39 (10)	C4A—C5A—H5AA	120.0
N2—C2—C3	113.91 (10)	C5A—C6A—C7A	119.87 (14)
C8—C3—C4	118.76 (10)	C5A—C6A—H6AA	120.1
C8—C3—C2	117.52 (10)	C7A—C6A—H6AA	120.1
C4—C3—C2	123.59 (10)	C6A—C7A—C2A	120.96 (13)
C5—C4—C3	120.45 (10)	C6A—C7A—H7AA	119.5
C5—C4—H4A	119.8	C2A—C7A—H7AA	119.5
C3—C4—H4A	119.8	C2B—C1B—S1B	104.01 (8)
C4—C5—C6	119.97 (10)	C2B—C1B—H1BA	111.0
C4—C5—H5A	120.0	S1B—C1B—H1BA	111.0
C6—C5—H5A	120.0	C2B—C1B—H1BB	111.0
O2—C6—C7	124.37 (10)	S1B—C1B—H1BB	111.0
O2—C6—C5	115.33 (10)	H1BA—C1B—H1BB	109.0
C7—C6—C5	120.30 (10)	C3B—C2B—C7B	119.71 (11)
C8—C7—C6	118.78 (10)	C3B—C2B—C1B	120.85 (10)
C8—C7—H7A	120.6	C7B—C2B—C1B	119.37 (10)
C6—C7—H7A	120.6	C4B—C3B—C2B	120.03 (12)
C7—C8—C3	121.73 (10)	C4B—C3B—H3BA	120.0
C7—C8—H8A	119.1	C2B—C3B—H3BA	120.0
C3—C8—H8A	119.1	C5B—C4B—C3B	120.10 (12)
O2—C9—H9A	109.5	C5B—C4B—H4BA	120.0
O2—C9—H9B	109.5	C3B—C4B—H4BA	120.0
H9A—C9—H9B	109.5	C4B—C5B—C6B	120.11 (12)
O2—C9—H9C	109.5	C4B—C5B—H5BA	119.9
H9A—C9—H9C	109.5	C6B—C5B—H5BA	119.9
H9B—C9—H9C	109.5	C7B—C6B—C5B	120.02 (12)
C2A—C1A—S1A	110.33 (8)	C7B—C6B—H6BA	120.0
C2A—C1A—H1AA	109.6	C5B—C6B—H6BA	120.0
S1A—C1A—H1AA	109.6	C6B—C7B—C2B	120.03 (11)
C2A—C1A—H1AB	109.6	C6B—C7B—H7BA	120.0
S1A—C1A—H1AB	109.6	C2B—C7B—H7BA	120.0
H1AA—C1A—H1AB	108.1		
			
C1—N1—N2—C2	−154.32 (10)	C2—C3—C8—C7	175.34 (10)
N2—N1—C1—S1A	−173.64 (7)	C1—S1A—C1A—C2A	154.36 (10)
N2—N1—C1—S1B	6.23 (13)	S1A—C1A—C2A—C7A	−101.53 (12)
C1A—S1A—C1—N1	5.25 (11)	S1A—C1A—C2A—C3A	79.84 (13)
C1A—S1A—C1—S1B	−174.63 (7)	C7A—C2A—C3A—C4A	−0.56 (17)
C1B—S1B—C1—N1	−174.67 (9)	C1A—C2A—C3A—C4A	178.07 (11)
C1B—S1B—C1—S1A	5.21 (8)	C2A—C3A—C4A—C5A	−0.08 (19)
N1—N2—C2—O1	−1.25 (17)	C3A—C4A—C5A—C6A	0.4 (2)
N1—N2—C2—C3	177.27 (9)	C4A—C5A—C6A—C7A	−0.1 (2)
O1—C2—C3—C8	−24.63 (15)	C5A—C6A—C7A—C2A	−0.55 (19)
N2—C2—C3—C8	156.84 (10)	C3A—C2A—C7A—C6A	0.88 (18)
O1—C2—C3—C4	151.22 (11)	C1A—C2A—C7A—C6A	−177.77 (11)
N2—C2—C3—C4	−27.31 (15)	C1—S1B—C1B—C2B	−173.50 (7)
C8—C3—C4—C5	0.03 (17)	S1B—C1B—C2B—C3B	−101.80 (11)
C2—C3—C4—C5	−175.77 (10)	S1B—C1B—C2B—C7B	74.98 (11)
C3—C4—C5—C6	0.60 (17)	C7B—C2B—C3B—C4B	0.15 (17)
C9—O2—C6—C7	2.19 (15)	C1B—C2B—C3B—C4B	176.93 (11)
C9—O2—C6—C5	−177.81 (10)	C2B—C3B—C4B—C5B	−0.74 (19)
C4—C5—C6—O2	179.43 (10)	C3B—C4B—C5B—C6B	0.37 (19)
C4—C5—C6—C7	−0.57 (17)	C4B—C5B—C6B—C7B	0.58 (19)
O2—C6—C7—C8	179.90 (10)	C5B—C6B—C7B—C2B	−1.16 (17)
C5—C6—C7—C8	−0.09 (16)	C3B—C2B—C7B—C6B	0.79 (16)
C6—C7—C8—C3	0.75 (17)	C1B—C2B—C7B—C6B	−176.03 (10)
C4—C3—C8—C7	−0.72 (17)		

### Hydrogen-bond geometry (Å, °)

**Table:**

*D*—H···*A*	*D*—H	H···*A*	*D*···*A*	*D*—H···*A*
C6A—H6AA···O1^i^	0.95	2.41	3.2516 (17)	147
C1B—H1BA···O1^ii^	0.99	2.35	3.2872 (16)	157
C3B—H3BA···O1^iii^	0.95	2.67	3.5351 (18)	152
C6B—H6BA···O2^iv^	0.95	2.47	3.4173 (17)	174

Symmetry codes: (i) −*x*+1, −*y*+1, −*z*+1; (ii) −*x*+1, −*y*+1, −*z*; (iii) *x*−1, *y*, *z*; (iv) −*x*+1, −*y*+2, −*z*−1.

## References

[bb1] Bharti, N., Maurya, M. R., Naqvi, F., Bhattcharya, A., Bhattacharya, S. & Azam, A. (2000). *Eur. J. Med. Chem.* **35**, 481–486.

[bb2] Boschi, A., Bolzati, C., Uccelli, L. & Duatti, A. (2003). *Nucl. Med. Biol.* **30**, 381–387. 10.1016/s0969-8051(03)00002-7 12767395

[bb3] Bruker (2006). *APEX2* and *SAINT* Bruker AXS Inc., Madison, Wisconsin, USA, 2006.

[bb4] Sheldrick, G. M. (1996). *SADABS* University of Göttingen, Germany.

[bb5] Sheldrick, G. M. (2008). *Acta Cryst.* A**64**, 112–122. 10.1107/S0108767307043930 18156677

[bb6] Singh, N. K., Singh, M. & Butcher, R. J. (2007). *Acta Cryst.* E**63**, o4405.

[bb7] Spek, A. L. (2009). *Acta Cryst.* D**65**, 148–155. 10.1107/S090744490804362X PMC2631630 19171970

